# Low-beta cortico-pallidal coherence decreases during movement and correlates with overall reaction time

**DOI:** 10.1016/j.neuroimage.2017.07.024

**Published:** 2017-10-01

**Authors:** Bernadette C.M. van Wijk, Wolf-Julian Neumann, Gerd-Helge Schneider, Tilmann H. Sander, Vladimir Litvak, Andrea A. Kühn

**Affiliations:** a Department of Neurology, Charité - University Medicine Berlin, Germany; b Wellcome Trust Centre for Neuroimaging, University College London, UK; c Department of Neurosurgery, Charité - University Medicine Berlin, Germany; d Physikalisch-Technische Bundesanstalt, Institut Berlin, Germany; e Berlin School of Mind and Brain, Charité - University Medicine Berlin, Germany; f NeuroCure, Charité - University Medicine Berlin, Germany

**Keywords:** Globus pallidus internus, Coherence, Beta oscillations, Dystonia, Movement, Magneto-encephalography

## Abstract

Beta band oscillations (13–30 Hz) are a hallmark of cortical and subcortical structures that are part of the motor system. In addition to local population activity, oscillations also provide a means for synchronization of activity between regions. Here we examined the role of beta band coherence between the internal globus pallidus (GPi) and (motor) cortex during a simple reaction time task performed by nine patients with idiopathic dystonia. We recorded local field potentials from deep brain stimulation (DBS) electrodes implanted in bilateral GPi in combination with simultaneous whole-head magneto-encephalography (MEG). Patients responded to visually presented go or stop-signal cues by pressing a button with left or right hand. Although coherence between signals from DBS electrodes and MEG sensors was observed throughout the entire beta band, a significant movement-related decrease prevailed in lower beta frequencies (∼13–21 Hz). In addition, patients' absolute coherence values in this frequency range significantly correlated with their median reaction time during the task (*r* = 0.89, *p* = 0.003). These findings corroborate the recent idea of two functionally distinct frequency ranges within the beta band, as well as the anti-kinetic character of beta oscillations.

## Highlights

• Simultaneous internal pallidum LFP and MEG recordings in dystonia patients.
• Cortico-pallidal coherence was found throughout the beta frequency range.
• Predominantly low-beta coherence (13–21 Hz) decreased with movement.
• Overall level of coherence was indicative of subject's median reaction time.
• No correlations were found between beta coherence measures and clinical scores.

## 1 Introduction

The importance of basal ganglia structures in controlling movement has been demonstrated by the success of deep brain stimulation in the treatment of movement disorders (Kleiner-Fisman et al., 2006, Perlmutter and Mink, 2006). Local field potential recordings from these electrodes in target structures such as the subthalamic nucleus (STN) (Cassidy et al., 2002; Kühn et al., 2004; Alegre et al., 2005, Androulidakis et al., 2007b, Cassidy et al., 2002, Kühn et al., 2004, Litvak et al., 2012), globus pallidus internus (GPi) (Brücke et al., 2008, Tsang et al., 2012, Talakoub et al., 2016), and ventral lateral thalamus (including Vim) (Paradiso et al., 2004, Klostermann et al., 2007, Brücke et al., 2013) have revealed movement-related modulations in the amplitude of beta (13–30 Hz) and gamma (∼40–90 Hz) oscillations that are strikingly similar to those found in motor cortex. This suggests that neural activity throughout the cortical-basal ganglia-thalamus network is closely coordinated.

Beta band coherence in rest recordings has been reported between the STN and motor cortex (Hirschmann et al., 2011, Litvak et al., 2011a), and between STN and GPi (Brown et al., 2001) in Parkinson's disease patients, and between GPi and motor cortex in dystonia patients (Neumann et al., 2015). Although spectral beta power is clearly reduced during movement, it is less clear whether beta coherence follows the same pattern. Some studies report a decrease in STN-cortical coherence with movement (Kühn et al., 2006a, Lalo et al., 2008, Alegre et al., 2010) but others failed to observe a significant effect (Litvak et al., 2012, Hirschmann et al., 2013). This discrepancy might be related to the recent notion of a functional subdivision in the beta band into a low and high frequency range. Whereas Parkinsonian symptoms of bradykinesia and rigidity are associated with low-beta power in the STN (Litvak et al., 2011a, Neumann et al., 2016, van Wijk et al., 2016), disease-unrelated coherence between STN and motor cortex was reported predominantly for high-beta frequencies in the same studies. When low-beta STN-cortex coherence is present, it is often of a slightly more lateral cortical location than the mesial high-beta coherence (Toledo et al., 2014, Oswal et al., 2016). It might therefore be relevant to look more closely at which beta frequencies experimental effects occur when interpreting results.

As the main output structure of the basal ganglia, the GPi is of special interest in studying the influence of basal ganglia on movement initiation. In our previous work involving dystonia patients (Neumann et al., 2015) we identified three sources of cortico-pallidal connectivity that were spatially distinct and frequency specific. In the theta range (4–8 Hz) pallidal activity was mainly coherent with temporal cortical regions; in the alpha range (7–13 Hz) with the cerebellum, and in the beta range (13–30 Hz) with sensorimotor regions. Only the pallido-cerebellar alpha coherence showed an inverse correlation with severity of dystonic symptoms. This leaves the possibility that the other networks are not disease-related but inherent to normal brain physiology. As these networks were found in rest recordings, it remains to be tested whether they have functional correlates, such as the control of movement.

The aim of this study was to investigate whether beta band cortico-pallidal coherence significantly reduces during movement, and whether it shows evidence of a subdivision into a low- and high-beta range. In addition, we correlated coherence values with measures of reaction time and clinical scores, in order to test for a link with behavior or disease severity. Results substantiate desynchronization in the low-beta band as a prerequisite for fast motor performance, and add to a more complete picture of cortical-basal ganglia oscillation patterns and the anti-kinetic role of beta band synchronization.

## 2 Methods

### 2.1 Patients and surgery

Nine patients (six female) with either segmental (*n* = 2), cervical (*n* = 4), Meige Syndrome (*n* = 1) or generalized dystonia (*n* = 2) took part in this study. 8 of them also participated in the rest recordings presented in Neumann et al. (2015); all except case 3. Patient characteristics are summarized in Table 1. Their mean age at time of recording was 51.4 (±12.1 SD) years, with an average disease duration of 14.0 (±6.7 SD) years. All patients were right-handed by self-report. Severity of clinical symptoms was assessed via the Toronto Western Spasmodic Torticollis Rating Scale (TWSTRS) for patients with cervical or segmental dystonia and the Burke Fahn Marsden Dystonia Rating scale (BFMDRS) for patients with generalized dystonia. All patients were implanted with deep brain stimulation (DBS) macroelectrodes (model 3389, Medtronic, Minneapolis, MN, USA) in left and right globus pallidus internus. Each electrode lead had four contact points with a diameter of 1.27 mm, length of 1.5 mm and an inter-contact spacing of 2 mm centre-to-centre. Correct placement of the DBS electrodes was guided by intraoperative microelectrode recordings and confirmed by postoperative MRI (Neumann et al., 2015).

**Table 1:** Patient characteristics. Toronto Western Spasmodic Torticollis Rating Scale (TWSTRS) was used for patients with cervical or segmental dystonia and the Burke Fahn Marsden Dystonia Rating scale (BFMDRS) in generalized dystonia (indicated with an *). Median reaction time was taken across the entire experiment, hence combining left and right hand trials. Likewise, average absolute coherence in the low-beta range (13–21 Hz) was averaged across left and right hemispheres.

Case	Age	Gender	Diagnosis	Preoperative TWSTRS/BFMDRS*	Disease duration (years)	% Correct go-trials	Median reaction time (s)	Average absolute low-beta coherence
1	48	F	Generalized Dystonia	16*	20	98	0.98	0.010
2	55	M	Segmental Dystonia	20	12	68	1.20	0.016
3	52	F	Meige Syndrome	na	15	51	0.65	0.002
4	51	F	Cervical Dystonia	23	3	97	0.69	0.003
5	52	F	Cervical Dystonia	22	11	82	0.91	0.005
6	48	F	Segmental Dystonia	25	6	100	0.66	0.006
7	68	M	Cervical Dystonia	16	23	94	0.65	0.007
8	58	M	Cervical Dystonia	18	20	88	0.92	0.009
9	24	F	Generalized Dystonia (DYT1)	27*	16	74	0.48	0.002

### 2.2 Recordings and experimental task

Recordings took place off medication and between 1 and 7 days after implantation while electrode leads were still externalized. Pallidal local field potential (LFP) recordings were obtained simultaneously with 125 channel magneto-encephalography (MEG, Yokogawa ET 160) at the Physikalisch Technische Bundesanstalt in Berlin. All patients were informed of the experimental procedures and gave written informed consent prior to the recordings. The study was approved by the local ethics committee of the Charité - University Medicine Berlin, Campus Virchow Klinikum, and was conducted in accordance with the declaration of Helsinki. Recordings for the stop-signal task included in this study took place after the 3–4 min rest recordings presented in Neumann et al. (2015) in the same session.

Stimuli were presented in black against a white background on a screen in front of the subjects. An experimental block comprised a mixture of left and right hand ‘go’ and ‘stop’ trials presented in a random order. Each trial started with a fixation cross displayed with a random duration between 4 and 6s. This was followed by presentation of a cue in the form of a ‘<’ or ‘>’-sign indicating the response hand. Subjects were instructed to press a button with the corresponding left or right index finger as soon as the cue appeared. They were told to withhold their response when a red box appeared around the cue (‘stop’). Each block contained 100 trials in total, of which 66% were ‘go’ and 33% ‘stop’ trials. Subjects completed at least one block and performed (part of) a second one depending on their level of fitness. Signals were low-pass filtered with 250 Hz and sampled at a rate of 2000 Hz. LFP signals were off-line converted to a bipolar montage between adjacent contact pairs.

### 2.3 Data analysis

We focused our analysis on the time window around the button press after the go-cues. Stop signal related changes were not analyzed due to limited number of trials and technical shortcomings for stop signal recording. Only go-trials in which subjects responded with the correct hand between 100 and 2000 ms after cue presentation were included. After applying notch filters at 50 Hz and higher-order harmonics (5th order bi-directional Butterworth filter with cut-off frequencies ±2 Hz), and downsampling to 1000 Hz, continuous time series were epoched from -3.5s until 3.5s around each button press (at 0s). Trials containing LFP amplitude values exceeding 7 standard deviations of the trial's time series were discarded. This resulted on average in 29 trials per left or right hand condition (range 18–44). For one patient (case 9) we were only able to record the right hand condition due to temporary technical failure of the left hand button. This patient was also the only subject not showing evidence of distinct LFP-MEG beta band coherence in either left or right hemisphere, likely due to the presence of strong wire-induced artefacts across all MEG channels, and was left out of the analysis altogether. All reported results are hence based on 8 subjects (case 1–8).

Although we experienced artefacts in the MEG due to the remanent magnetization of the percutaneous connection wires, coherence estimates between LFP and MEG signals on the sensor level seemed largely unaffected. This is probably related to the frequency band of interest (>10 Hz), which is above pathology-induced involuntary body movements leading to minuscule head position jitter. We only excluded one channel that systematically exhibited bad recordings across sessions. Previously we have shown that cortico-pallidal beta band coherence can be localized to sensorimotor areas in the same group of patients (Neumann et al., 2015). For the current study we conducted our analyses at sensor level. Raw MEG data were converted and epoched using SPM12 (Litvak et al., 2011b) but actual computations were done using custom Matlab scripts. First, we computed coherence for each LFP channel and all MEG sensors individually in the range of -3s to 3s. For this, auto- and cross-spectral densities were estimated using Welch's method with 50% overlapping 250 ms Hanning windows. Fieldtrip's function ft_topoplotTFR.m (Oostenveld et al., 2011) was used for visualization of the topographies in the a-priori chosen beta frequency range (13–30 Hz) (Engel and Fries, 2010, van Wijk et al., 2012, Brittain and Brown, 2014) averaged across left and right hand conditions. We selected for each LFP channel the five MEG sensors with largest coherence values while avoiding sensors on the edge of the helmet as these are more likely to be contaminated by muscle artefacts.

Subsequently, time-resolved coherence spectra were computed for the selected LFP-MEG channel combinations with 750 ms sliding time windows in steps of 100 ms. Movement-related changes in coherence were assessed by expressing the time-resolved coherence spectra as a percentage change compared to the average coherence in the -3 to -1s (pre-movement) time window for each frequency. Resulting spectra were averaged across all selected LFP-MEG channels combinations per hemisphere and across conditions, resulting in separate spectra for the hemisphere contralateral and ipsilateral to the moving hand as well as an average across hemispheres. Likewise, we computed time-resolved power spectra for all bipolar LFP channels and selected MEG channels (based on coherence) using identical settings and averaging. For the power spectra, frequencies between 47 and 53 were removed from the time-frequency spectra and linearly interpolated. We also used robust averaging (Wager et al., 2005) with offset value 7 to reduce the influence of bad data segments.

### 2.4 Statistics

We tested for significant changes in the movement-related coherence and power spectra using SPM12 (Litvak et al., 2011b). Individual spectra were transformed into images and used for second level statistical analysis. This involved a one sample *t*-test against 0 (no change from baseline) for each time-frequency sample in the spectrum. We narrowed our window to frequencies between 8 and 90 Hz. To protect against the multiple comparisons problem, we only considered clusters of samples that survived a False Discovery Rate (FDR) correction of *p* < .05. We mildly smoothed individual's power spectra (full width half maximum of 200 ms and 2 Hz) before applying statistics in order to reduce irregular cluster edges due to robust averaging.

Furthermore, we tested for Pearson's correlations between coherence values and TWSTRS scores (when available) and between coherence values and median reaction time. This was done for absolute coherence values averaged across either low-beta (13–21 Hz) or high-beta (21–30 Hz) frequencies and taken from the entire -3 to 3s time interval, -3 to -1s (pre-movement), -1 to 1s (movement) or 1 to 3s (post-movement). Movement-related coherence values were also considered by expressing the values during the movement time period as a percentage change from the pre-movement time period. The subdivision of the beta band into these low- and high-frequency ranges was based on previous work (Priori et al., 2004, Little et al., 2013b, Oswal et al., 2016, van Wijk et al., 2016). For each correlation, one value per subject entered the analysis (conditions and hemispheres were averaged). Correlations with median reaction time were also performed by separating left and right hemispheres, hence entering two coherence values and corresponding contralateral median reaction time per subject.

To test whether findings for coherence were independent of spectral power, we repeated the correlations with median reaction time and clinical scores for absolute and movement-related power in the GPi and cortex. We also correlated the movement-related decrease in beta coherence with movement-related decreases in power across subjects. Kolmogorov-Smirnov tests indicated that none of the variables significantly differed from a normal distribution. All correlation *p*-values remained significant after correcting for the FDR for independent or positively dependent tests (Benjamini and Yekutieli, 2001) per outcome variable and measure (coherence or power) unless reported otherwise.

## 3 Results

### 3.1 Coherence topographies and spectra

**Fig. 1:**
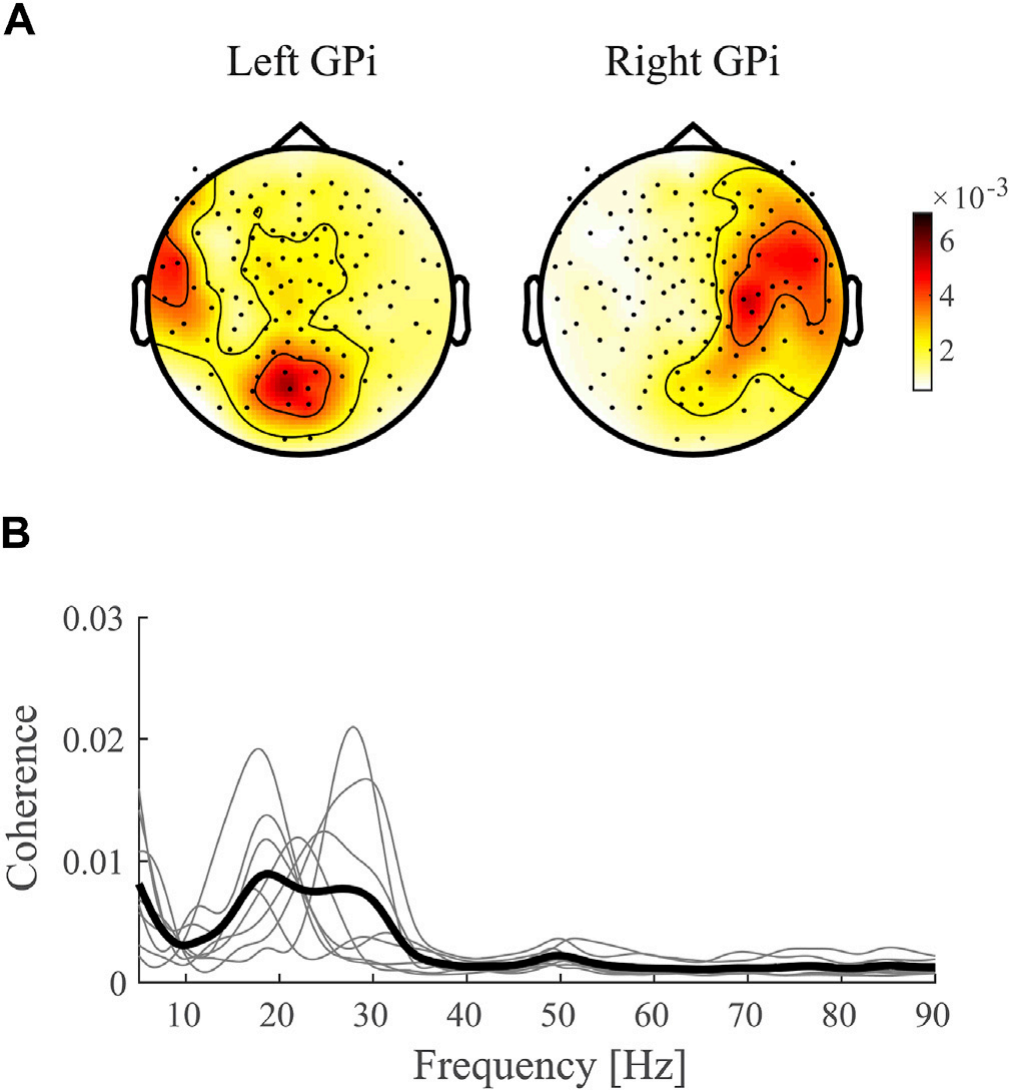
**Grand-average coherence. A)** Topographies of beta coherence (13–30 Hz) for left and right GPi separately. Shown is the average across the -3 to 3s time interval and all subjects. Red colors indicate larger coherence values. **B)** Coherence spectra for each individual subject (thin lines) and the grand average (thick line). Spectra are averaged across selected LFP-MEG channel combinations, hemispheres, and conditions.

Cortico-pallidal beta coherence was clearly lateralized to the ipsilateral hemisphere (Fig. 1A). Although coherence with the right GPi peaked for MEG sensors above central areas, coherence with the left GPi was located more posterior. The discrepancy between the topographies might be related to artefacts arising from the percutaneous wires that were located on the left side of the head (F3 in the 20/20 system). In addition, the subject's head was not always exactly aligned on the midline due to their dystonic symptoms. Nevertheless, coherence for the left GPi could be clearly detected and showed similar movement-related changes as the right GPi. No difference was found between left and right hemisphere absolute beta coherence values (paired-samples *t*-test *t*(7) = 0.003, *p* = 0.998). Likewise, median reaction times for right hand movements did not differ significantly from left hand movements (*t*(7) = 0.052, *p* = 0.960). For that reason, we combined left and right hemispheres into contra- and ipsilateral conditions with respect to the moving hand, or a single average per subject. A distinct coherence peak was identified in all 8 subjects included in the analysis with a peak frequency that could occur throughout the entire beta frequency range (Fig. 1B).

### 3.2 Movement-related changes in coherence and power

**Fig. 2:**
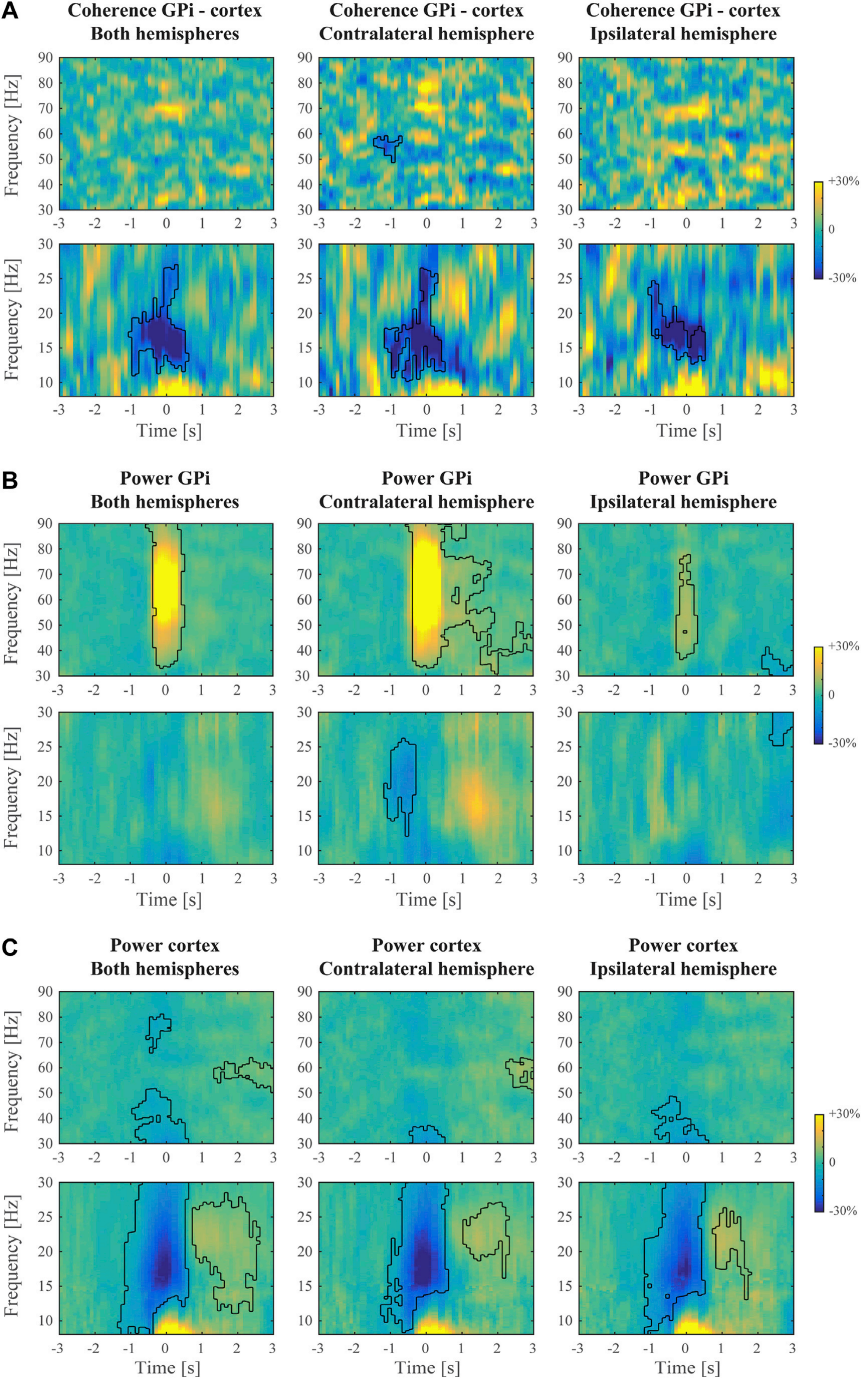
**Movement-related coherence and power spectra.** Shown are the grand-average changes in A) cortico-pallidal coherence; B) pallidal power; and C) cortical power, expressed as a percentage of the average in the -3 to -1s pre-movement time window. Left and right hand conditions were combined to form contralateral and ipsilateral hemispheres with respect to the moving hand. Significant clusters under FDR-corrected cluster-level *p* < .05 are outlined with black contour lines. For better visibility, we show beta and gamma frequency ranges in different panels but note that we performed the FDR correction on the entire spectrum. No significant beta coherence clusters were detected when contrasting ipsilateral and contralateral spectra.

Movement-related changes in coherence are shown in Fig. 2A. Significant clusters under an FDR correction of *p* < .05 are outlined. These occurred around movement onset and primarily for frequencies in the low-beta range. Significant decreases for high-beta frequencies were also observed but these were expressed much briefer in time. Results for ipsilateral and contralateral hemispheres were strikingly similar, albeit with a somewhat smaller significant cluster for the ipsilateral hemisphere. No significant cluster in the beta range was detected when directly contrasting ipsi- and contralateral spectra. A decrease in (low-)beta coherence hence occurred independent of which hand was moving. Increases in gamma band coherence were rather scattered across different frequencies and not found to be significant on group-level.

**Fig. 3:**
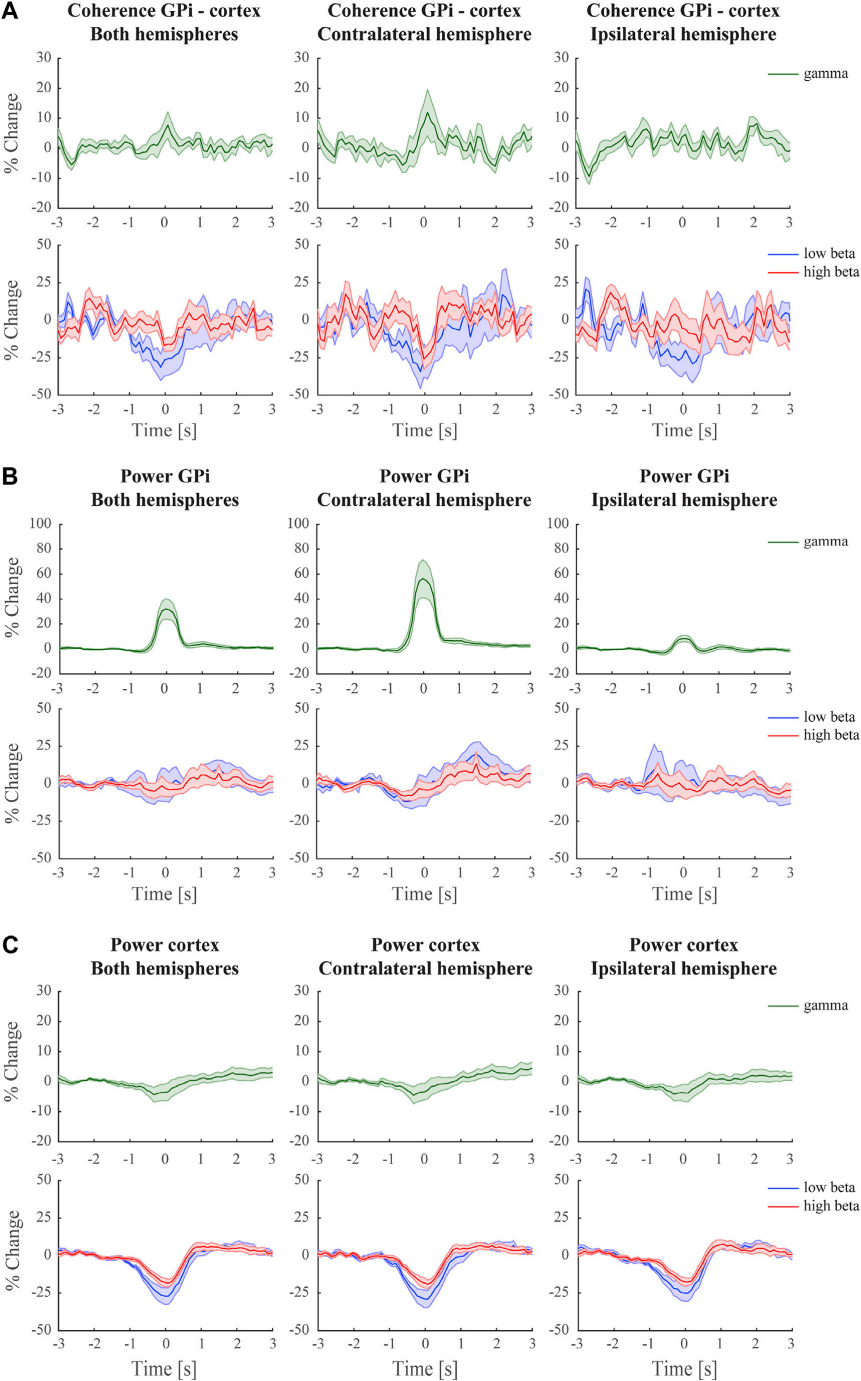
**Movement-related coherence and power per frequency (sub)band.** This figure shows the time-frequency modulations as presented in Fig. 2 averaged within the gamma (30–90 Hz), low-beta (13–21 Hz), and high-beta (21–30 Hz) range. A) Cortico-pallidal coherence; B) pallidal power; C) cortical power. Shaded bars indicate the standard error of the mean across subjects.

By contrast, the movement-related increase in pallidal gamma power was well pronounced and lateralized to the contralateral hemisphere (Fig. 2B). Beta suppression was present in both low- and high-frequency ranges but was relatively weak. The modulations found for cortical power more resembled those for coherence, with strongest beta suppression for low-beta frequencies (Fig. 2C) and no clear gamma increase was observed. For better comparison between frequency (sub)band-specific modulations we further illustrate these findings as line plots in Fig. 3.

### 3.3 Correlations with reaction time and clinical score

**Fig. 4:**
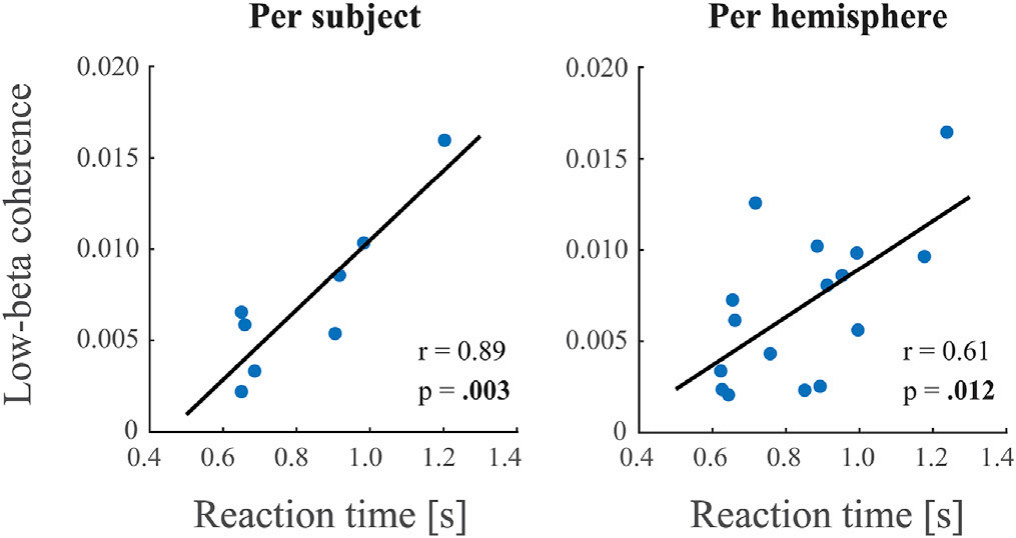
**Correlations between low-beta coherence and reaction time.** Shown here are the significant correlations for low-beta coherence (13–21 Hz) in the entire -3 to 3s time window. Reaction times were subjects' median reaction time across the experiment (left panel) or the median reaction time per response hand (right panel). Likewise, coherence values were averaged across hemispheres (left panel) or taken separately per hemisphere (right panel).

Low-beta coherence (13–21 Hz) showed significant correlations with reaction time. The strongest correlation was found for a subject's average coherence value in the entire -3 to 3s time window and their median reaction time across the experiment (*r* = 0.89, *p* = 0.003). However, significant correlations were also found in task-specific time intervals (pre-movement: *r* = 0.87, *p* = 0.005; movement: *r* = 0.82, *p* = 0.013; post-movement: *r* = 0.77, *p* = 0.025 (not significant under FDR correction)). Interestingly, the movement-related decrease was not related to reaction time (*r* = 0.26, *p* = 0.538). Also no significant correlations (*p* > .24) were found for the high-beta range (21–30 Hz). Analyses repeated with left and right hemisphere as separate entries resulted in similar but slightly weaker findings (entire epoch: *r* = 0.61, *p* = 0.012; pre-movement: *r* = 0.62, *p* = 0.011; movement: *r* = 0.65, *p* = 0.007; post-movement: *r* = 0.31, *p* = 0.243). Again with no significant findings for the movement-related decrease or the high-beta range (*p* > .34). Results for low-beta coherence in the -3 to 3s time window are depicted in Fig. 4 and listed in Table 1.

Significant correlations with reaction time were not found for pallidal or cortical power, neither for absolute nor movement-related low- or high-beta frequency ranges (*p* > .14). Moreover, subjects' average movement-related suppression of beta coherence was not found to be correlated with their average suppression in pallidal or cortical power, neither in low- nor high-beta ranges (*p* > .33). Cortico-pallidal coherence hence seems to contribute to task performance independently from spectral power. As in our previous study (Neumann et al., 2015) we did not find a significant correlation between any beta coherence (*p* > .21) or power (*p* > .16) values and severity of clinical symptoms.

## 4 Discussion

We showed that cortico-pallidal beta-band coherence in dystonia patients significantly decreases before and during movement. Although individual peaks were distributed across the entire beta range, mainly low-beta frequencies (13–21 Hz) showed a significant decrease with movement. Moreover, absolute coherence values in this frequency range were positively correlated with subjects' reaction time, independent of time window. A significant decrease in high-beta (21–30 Hz) coherence was confined to a brief period around movement onset, and no correlations were found with reaction time. Although pallidal and cortical beta power were also suppressed with movement, the magnitude of this suppression was found to be unrelated to that of coherence. In addition, correlations with reaction time were unique for coherence. Our findings therefore suggest that cortico-pallidal coherence contributes to the movement task independently from spectral power.

Both the movement-related decrease and positive correlation with reaction time underscore the anti-kinetic role of beta oscillations (Kühn et al., 2004, Brown and Williams, 2005). This role not only applies to locally synchronized activity but could be extended to inter-regional synchronization, as for example seen for cortico-spinal coherence (Androulidakis et al., 2007a, van Wijk et al., 2009). The anti-kinetic character could be manifest as an increase in beta amplitude in anticipation to upcoming postural perturbations (Gilbertson et al., 2005, Androulidakis et al., 2007a), prolonged reaction times when movements are initiated during time periods with prominent beta oscillations (Zhang et al., 2008, Matsuya et al., 2013, Mcallister et al., 2013), or slower execution of the response (Joundi et al., 2012, Chen et al., 2007, Gilbertson et al., 2005, Pogosyan et al., 2009). Moreover, the relation between elevated beta power in the subthalamic nucleus and severity of bradykinesia and rigidity in Parkinson's disease is well established (Weinberger et al., 2006, Kühn et al., 2006b, Kühn et al., 2008, Kühn et al., 2009, Eusebio et al., 2011, Ray et al., 2012, Neumann et al., 2016).

Only absolute coherence values were significantly correlated with reaction time. Even during the -1 to 1s time window around movement onset, subjects' absolute coherence and not the relative movement-related decrease was indicative of the reaction time. Although other movement parameters such as velocity were not acquired in our study, our results suggest that task performance relates to the overall beta band coherence within the motor network. Reduction in beta band coherence starts well before the movement and thus may be a prerequisite for movements to occur. It could be speculated that reduced beta band coherence allows other frequencies such as gamma band activity to occur that are more specifically related to parameters of motor performance (Brücke et al., 2012). Previous electrophysiological studies have found the amplitude and velocity of performed movements to be reflected in the size of induced GPi gamma band synchronization (Brücke et al., 2012, Singh and Bötzel, 2013). The magnitude of beta power attenuation could also reflect movement velocity (Singh and Bötzel, 2013) as well as sequence boundaries within continuous movements (Herrojo Ruiz et al., 2014). Also fMRI studies indicate movement parameters to be encoded within the GPi (Vaillancourt et al., 2007, Vaillancourt et al., 2004, Spraker et al., 2007). Further studies with a more specific experimental design are desired to explore the relation between coherence and motor performance in more detail.

The notion of a functional subdivision of the beta band into a low- and high-frequency range stems from findings in Parkinson's disease. Severity of bradykinesia and rigidity seems closely linked to enhanced activity specifically in the low-beta range (Little et al., 2013a, van Wijk et al., 2016) whereas high-beta has been linked to freezing of gait (Toledo et al., 2014). Subtle differences in cortical topography and estimates of time delays underlying STN-cortical beta coherence hint at a contribution of the indirect pathway to low-beta oscillations and the hyperdirect pathway for high-beta oscillations (Oswal et al., 2016). If the neuronal origin of low- and high-beta oscillations could indeed be pinpointed to these distinct pathways, this would imply at least partly separable mechanisms that might work conjointly to facilitate or inhibit motor actions. Considering these frequency ranges separately in experimental studies may reveal their specific contributions.

As the main output structure of the basal ganglia, the internal pallidum is where indirect, direct, and hyperdirect pathways converge before inhibiting the thalamus. It is hence likely to relay imperative information regarding movement selection and initiation. This underscores the value of experimental studies with DBS electrodes implanted in GPi, typically for dystonia treatment, also for fundamental research. On the other hand, one should be cautious when drawing conclusions on normal brain neurophysiology from patient recordings. Many previous studies on beta oscillations in subcortical structures have been conducted in the context of Parkinson's disease. Some of the inter-regional beta synchronization could only be detected in the Parkinsonian state (Sharott et al., 2005) or shows a strong suppression with dopaminergic medication (Brown et al., 2001, Hirschmann et al., 2013, Little et al., 2013b). Beta coherence between regions of the motor system is also a common feature in healthy individuals (van Wijk et al., 2012). It remains therefore challenging to determine to what extent oscillatory activity picked up with DBS electrodes in patients is normal or pathological.

Neither absolute nor movement-related beta coherence was found to be correlated with symptom severity in the present study. This is in line with our previous work where we only found a significant relation for alpha coherence between GPi and cerebellum (Neumann et al., 2015). Unlike Parkinson's disease, dystonic syndromes are hyperkinetic disorders that are characterized by twisting movements and abnormal posture resulting from involuntary sustained and sometimes repetitive muscle contractions (Fahn, 1988). Although our sample size was rather small (only six patients could be included in the correlation analysis), dystonia is presumably more associated with increased power for frequencies below the beta band (Silberstein et al., 2003, Chen et al., 2006, Liu et al., 2008, Sharott et al., 2008). Decreased and more irregular neuronal firing in GPi is distinctive of the disorder, possible caused by abnormal striatal activity (Hendrix and Vitek, 2012). Deep brain stimulation might be effective by suppressing pathological 4–12 Hz oscillations (Barow et al., 2014). Unfortunately, our sample size was also too small to draw conclusions about dystonia subtypes and coherence magnitude. Furthermore, it is possible that the heterogeneity of the group might have masked (subtype-specific) associations with clinical parameters.

In conclusion, our findings revealed the involvement of predominantly low-beta cortico-pallidal coherence in movement execution and initiation. A significant movement-related attenuation of low-beta coherence was found in both contralateral and ipsilateral hemispheres. Patients with strong absolute low-beta coherence values demonstrated slower overall reaction times. The lack of significant correlations with severity of dystonic symptoms suggest that these phenomena are intrinsic to normal brain physiology.

## References

[bib1] Alegre M., Alonso-Frech F., Rodríguez-Oroz M.C., Guridi J., Zamarbide I., Valencia M., Manrique M., Obeso J.A., Artieda J. (2005). Movement-related changes in oscillatory activity in the human subthalamic nucleus: ipsilateral vs. contralateral movements. Eur. J. Neurosci..

[bib2] Alegre M., Rodríguez-Oroz M.C., Valencia M., Pérez-Alcázar M., Guridi J., Iriarte J., Obeso J.A., Artieda J. (2010). Changes in subthalamic activity during movement observation in Parkinson's disease: is the mirror system mirrored in the basal ganglia?. Clin. Neurophysiol..

[bib3] Androulidakis A.G., Doyle L.M.F., Yarrow K., Litvak V., Gilbertson T.P., Brown P. (2007). Anticipatory changes in beta synchrony in the human corticospinal system and associated improvements in task performance. Eur. J. Neurosci..

[bib4] Androulidakis A.G., Kühn A.A., Chen C.C., Blomstedt P., Kempf F., Kupsch A., Schneider G.-H., Doyle L., Dowsey-Limousin P., Hariz M.I., Brown P. (2007). Dopaminergic therapy promotes lateralized motor activity in the subthalamic area in Parkinson's disease. Brain.

[bib5] Barow E., Neumann W.J., Brücke C., Huebl J., Horn A., Brown P., Krauss J.K., Schneider G.H., Kühn A.A. (2014). Deep brain stimulation suppresses pallidal low frequency activity in patients with phasic dystonic movements. Brain.

[bib6] Benjamini Y., Yekutieli D. (2001). The control of the False Discovery Rate in multiple testing under dependency. Ann. Stat..

[bib7] Brittain J.S., Brown P. (2014). Oscillations and the basal ganglia: motor control and beyond. Neuroimage.

[bib8] Brown P., Oliviero A., Mazzone P. (2001). Dopamine dependency of oscillations between subthalamic nucleus and pallidum in Parkinson's disease. J. Neurosci..

[bib9] Brown P., Williams D. (2005). Basal ganglia local field potential activity: character and functional significance in the human. Clin. Neurophysiol..

[bib10] Brücke C., Bock A., Huebl J., Krauss J.K., Schönecker T., Schneider G.H., Brown P., Kühn A.A. (2013). Thalamic gamma oscillations correlate with reaction time in a Go/noGo task in patients with essential tremor. Neuroimage.

[bib11] Brücke C., Huebl J., Schönecker T., Neumann W., Yarrow K., Kupsch A., Blahak C., Lu G., Brown P., Krauss J.K., Schneider G., Kühn A.A. (2012). Scaling of movement is related to pallidal gamma oscillations in patients with dystonia. J. Neurosci..

[bib12] Brücke C., Kempf F., Kupsch A., Schneider G.H., Krauss J.K., Aziz T., Yarrow K., Pogosyan A., Brown P., Kühn A.A. (2008). Movement-related synchronization of gamma activity is lateralized in patients with dystonia. Eur. J. Neurosci..

[bib13] Cassidy M., Mazzone P., Oliviero A., Insola A., Tonali P., Di Lazzaro V., Brown P. (2002). Movement-related changes in synchronization in the human basal ganglia. Brain.

[bib14] Chen C.C., Kühn A.A., Trottenberg T., Kupsch A., Schneider G.H., Brown P. (2006). Neuronal activity in globus pallidus interna can be synchronized to local field potential activity over 3-12 Hz in patients with dystonia. Exp. Neurol..

[bib15] Chen C.C., Litvak V., Gilbertson T., Kühn A., Lu C., Lee S., Tsai C., Tisch S., Limousin P., Hariz M. (2007). Excessive synchronization of basal ganglia neurons at 20 Hz slows movement in Parkinson's disease. Exp. Neurol..

[bib16] Engel A.K., Fries P. (2010). Beta-band oscillations – signalling the status quo?. Curr. Opin. Neurobiol..

[bib17] Eusebio A., Thevathasan W., Doyle Gaynor L., Pogosyan A., Bye E., Foltynie T., Zrinzo L., Ashkan K., Aziz T., Brown P. (2011). Deep brain stimulation can suppress pathological synchronisation in parkinsonian patients. J. Neurol. Neurosurg. Psychiatry.

[bib18] Fahn S. (1988). Concept and classification of dystonia. Adv. Neurol..

[bib19] Gilbertson T., Lalo E., Doyle L., Lazzaro V., Di Cioni B., Brown P. (2005). Existing motor state is favored at the expense of new movement during 13-35 Hz oscillatory synchrony in the human corticospinal system. J. Neurosci..

[bib20] Hendrix C.M., Vitek J.L. (2012). Toward a network model of dystonia. Ann. N. Y. Acad. Sci..

[bib21] Herrojo Ruiz M., Brücke C., Nikulin V.V., Schneider G., Kühn A.A. (2014). Beta-band amplitude oscillations in the human internal globus pallidus support the encoding of sequence boundaries during initial sensorimotor sequence learning. Neuroimage.

[bib22] Hirschmann J., Özkurt T.E., Butz M., Homburger M., Elben S., Hartmann C.J., Vesper J., Wojtecki L., Schnitzler A. (2013). Differential modulation of STN-cortical and cortico-muscular coherence by movement and levodopa in Parkinson's disease. Neuroimage.

[bib23] Hirschmann J., Özkurt T.E., Butz M., Homburger M., Elben S., Hartmann C.J., Vesper J., Wojtecki L., Schnitzler A. (2011). Distinct oscillatory STN-cortical loops revealed by simultaneous MEG and local field potential recordings in patients with Parkinson's disease. Neuroimage.

[bib24] Joundi R.A., Jenkinson N., Brittain J.-S., Aziz T.Z., Brown P. (2012). Driving oscillatory activity in the human cortex enhances motor performance. Curr. Biol..

[bib25] Kleiner-Fisman G., Herzog J., Fisman D.N., Tamma F., Lyons K.E., Pahwa R., Lang A.E., Deuschl G. (2006). Subthalamic nucleus deep brain stimulation: summary and meta-analysis of outcomes. Mov. Disord..

[bib26] Klostermann F., Nikulin V.V., Kühn A.A., Marzinzik F., Wahl M., Pogosyan A., Kupsch A., Schneider G.H., Brown P., Curio G. (2007). Task-related differential dynamics of EEG alpha- and beta-band synchronization in cortico-basal motor structures. Eur. J. Neurosci..

[bib27] Kühn A.A., Doyle L., Pogosyan A., Yarrow K., Kupsch A., Schneider G.-H., Hariz M.I., Trottenberg T., Brown P. (2006). Modulation of beta oscillations in the subthalamic area during motor imagery in Parkinson's disease. Brain.

[bib28] Kühn A.A., Kempf F., Brucke C., Gaynor Doyle L., Martinez-Torres I., Pogosyan A., Trottenberg T., Kupsch A., Schneider G.-H., Hariz M.I., Vandenberghe W., Nuttin B., Brown P. (2008). High-frequency stimulation of the subthalamic nucleus suppresses oscillatory activity in patients with Parkinson's disease in parallel with improvement in motor performance. J. Neurosci..

[bib29] Kühn A.A., Kupsch A., Schneider G.-H., Brown P. (2006). Reduction in subthalamic 8-35 Hz oscillatory activity correlates with clinical improvement in Parkinson's disease. Eur. J. Neurosci..

[bib30] Kühn A.A., Tsui A., Aziz T., Ray N., Brücke C., Kupsch A., Schneider G.-H., Brown P. (2009). Pathological synchronisation in the subthalamic nucleus of patients with Parkinson's disease relates to both bradykinesia and rigidity. Exp. Neurol..

[bib31] Kühn A.A., Williams D., Kupsch A., Limousin P., Hariz M., Schneider G.H., Yarrow K., Brown P. (2004). Event-related beta desynchronization in human subthalamic nucleus correlates with motor performance. Brain.

[bib32] Lalo E., Thobois S., Sharott A., Polo G., Mertens P., Pogosyan A., Brown P. (2008). Patterns of bidirectional communication between cortex and basal ganglia during movement in patients with Parkinson disease. J. Neurosci..

[bib33] Little S., Pogosyan A., Neal S., Zavala B., Zrinzo L., Hariz M., Foltynie T., Limousin P., Ashkan K., FitzGerald J., Green A.L., Aziz T.Z., Brown P. (2013). Adaptive deep brain stimulation in advanced Parkinson disease. Ann. Neurol..

[bib34] Little S., Tan H., Anzak A., Pogosyan A., Kühn A., Brown P. (2013). Bilateral functional connectivity of the basal ganglia in patients with Parkinson's disease and its modulation by dopaminergic treatment. PLoS One.

[bib35] Litvak V., Eusebio A., Jha A., Oostenveld R., Barnes G., Foltynie T., Limousin P., Zrinzo L., Hariz M.I., Friston K., Brown P. (2012). Movement-related changes in local and long-range synchronization in Parkinson's disease revealed by simultaneous magnetoencephalography and intracranial recordings. J. Neurosci..

[bib36] Litvak V., Jha A., Eusebio A., Oostenveld R., Foltynie T., Limousin P., Zrinzo L., Hariz M.I., Friston K., Brown P. (2011). Resting oscillatory cortico-subthalamic connectivity in patients with Parkinson's disease. Brain.

[bib37] Litvak V., Mattout J., Kiebel S., Phillips C., Henson R., Kilner J., Barnes G., Oostenveld R., Daunizeau J., Flandin G., Penny W., Friston K. (2011). EEG and MEG data analysis in SPM8. Comput. Intell. Neurosci..

[bib38] Liu X., Wang S., Yianni J., Nandi D., Bain P.G., Gregory R., Stein J.F., Aziz T.Z. (2008). The sensory and motor representation of synchronized oscillations in the globus pallidus in patients with primary dystonia. Brain.

[bib39] Matsuya R., Ushiyama J., Ushiba J. (2013). Prolonged reaction time during episodes of elevated β-band corticomuscular coupling and associated oscillatory muscle activity. J. Appl. Physiol..

[bib40] Mcallister C.J., Ro K.C., Stanford I.M., Woodhall G.L., Furlong P.L., Hall S.D. (2013). Oscillatory beta activity mediates neuroplastic effects of motor cortex stimulation in humans. J. Neurosci..

[bib41] Neumann W.-J., Degen K., Schneider G.-H., Brücke C., Huebl J., Brown P., Kühn A.A. (2016). Subthalamic synchronized oscillatory activity correlates with motor impairment in patients with Parkinson's disease. Mov. Disord..

[bib42] Neumann W., Jha A., Bock A., Huebl J., Horn A., Schneider G., Sander T.H., Litvak V., Kühn A.A. (2015). Cortico-pallidal oscillatory connectivity in patients with dystonia. Brain.

[bib43] Oostenveld R., Fries P., Maris E., Schoffelen J.-M. (2011). FieldTrip: open source software for advanced analysis of MEG, EEG, and invasive electrophysiological data. Comput. Intell. Neurosci..

[bib44] Oswal A., Beudel M., Zrinzo L., Limousin P., Hariz M., Foltynie T., Litvak V., Brown P. (2016). Deep brain stimulation modulates synchrony within spatially and spectrally distinct resting state networks in Parkinson's disease. Brain.

[bib45] Paradiso G., Cunic D., Saint-Cyr J.A., Hoque T., Lozano A.M., Lang A.E., Chen R. (2004). Involvement of human thalamus in the preparation of self-paced movement. Brain.

[bib46] Perlmutter J.S., Mink J.W. (2006). Deep brain stimulation. Annu. Rev. Neurosci..

[bib47] Pogosyan A., Gaynor L.D., Eusebio A., Brown P. (2009). Boosting cortical activity at beta-band frequencies slows movement in humans. Curr. Biol..

[bib48] Priori A., Foffani G., Pesenti A., Tamma F., Bianchi A., Pellegrini M., Locatelli M., Moxon K., Villani R. (2004). Rhythm-specific pharmacological modulation of subthalamic activity in Parkinson's disease. Exp. Neurol..

[bib49] Ray N.J., Brittain J.S., Holland P., Joundi R.A., Stein J.F., Aziz T.Z., Jenkinson N. (2012). The role of the subthalamic nucleus in response inhibition: evidence from local field potential recordings in the human subthalamic nucleus. Neuroimage.

[bib50] Sharott A., Grosse P., Kühn A.A., Salih F., Engel A.K., Kupsch A., Schneider G.H., Krauss J.K., Brown P. (2008). Is the synchronization between pallidal and muscle activity in primary dystonia due to peripheral afferance or a motor drive?. Brain.

[bib51] Sharott A., Magill P.J., Harnack D., Kupsch A., Meissner W., Brown P. (2005). Dopamine depletion increases the power and coherence of β-oscillations in the cerebral cortex and subthalamic nucleus of the awake rat. Eur. J. Neurosci..

[bib52] Silberstein P., Kühn A.A., Kupsch A., Trottenberg T., Krauss J.K., Wöhrle J.C., Mazzone P., Insola A., Lazzaro V., Di Oliviero A., Aziz T., Brown P. (2003). Patterning of globus pallidus local field potentials differs between Parkinson's disease and dystonia. Brain.

[bib53] Singh A., Bötzel K. (2013). Globus pallidus internus oscillatory activity is related to movement speed. Eur. J. Neurosci..

[bib54] Spraker M.B., Yu H., Corcos D.M., Vaillancourt D.E. (2007). Role of individual basal ganglia nuclei in force amplitude generation. J. Neurophysiol..

[bib55] Talakoub O., Neagu B., Udupa K., Tsang E., Chen R., Popovic M.R., Wong W. (2016). Time-course of coherence in the human basal ganglia during voluntary movements. Sci. Rep..

[bib56] Toledo J.B., López-Azcárate J., Garcia-Garcia D., Guridi J., Valencia M., Artieda J., Obeso J., Alegre M., Rodriguez-Oroz M. (2014). High beta activity in the subthalamic nucleus and freezing of gait in Parkinson's disease. Neurobiol. Dis..

[bib57] Tsang E.W., Hamani C., Moro E., Mazzella F., Lozano A.M., Hodaie M., Yeh I.-J., Chen R. (2012). Movement related potentials and oscillatory activities in the human internal globus pallidus during voluntary movements. J. Neurol. Neurosurg. Psychiatry.

[bib58] Vaillancourt D.E., Mayka M.A., Thulborn K.R., Corcos D.M. (2004). Subthalamic nucleus and internal globus pallidus scale with the rate of change of force production in humans. Neuroimage.

[bib59] Vaillancourt D.E., Yu H., Mayka M.A., Corcos D.M. (2007). Role of the basal ganglia and frontal cortex in selecting and producing internally guided force pulses. Neuroimage.

[bib60] van Wijk B.C.M., Beek P.J., Daffertshofer A. (2012). Neural synchrony within the motor system: what have we learned so far?. Front. Hum. Neurosci..

[bib61] van Wijk B.C.M., Beudel M., Jha A., Oswal A., Foltynie T., Hariz M.I., Limousin P., Zrinzo L., Aziz T.Z., Green A.L., Brown P., Litvak V. (2016). Subthalamic nucleus phase-amplitude coupling correlates with motor impairment in Parkinson's disease. Clin. Neurophysiol..

[bib62] van Wijk B.C.M., Daffertshofer A., Roach N., Praamstra P. (2009). A role of beta oscillatory synchrony in biasing response competition?. Cereb. Cortex.

[bib63] Wager T.D., Keller M.C., Lacey S.C., Jonides J. (2005). Increased sensitivity in neuroimaging analyses using robust regression. Neuroimage.

[bib64] Weinberger M., Mahant N., Hutchison W.D., Lozano A.M., Moro E., Hodaie M., Lang A.E., Dostrovsky J.O. (2006). Beta oscillatory activity in the subthalamic nucleus and its relation to dopaminergic response in Parkinson's disease. J. Neurophysiol..

[bib65] Zhang Y., Wang X., Bressler S.L., Chen Y., Ding M. (2008). Prestimulus cortical activity is correlated with speed of visuomotor processing. J. Cogn. Neurosci..

